# Early sowing enhances genotypic performance in mitigating the risk of wheat blast-induced yield loss: evidence from a 23-year simulation study in Bangladesh

**DOI:** 10.3389/fpls.2025.1568461

**Published:** 2025-09-26

**Authors:** Timothy J. Krupnik, José M. C. Fernandes, Willingthon Pavan, Thiago Berton Ferreira, Diego N. L. Pequeno, Gerrit Hoogenboom, Virginia L. Covert, Khaled Hossain, Md. Harun-Or-Rashid, Rabiul Islam, Alexandre Zanatta, Sabine Stuerz, Akbar Hossain

**Affiliations:** ^1^ Sustainable Agrifood Systems Program, International Maize and Wheat Improvement Center (CIMMYT), Dhaka, Bangladesh; ^2^ Department of Plant Pathology, Brazilian Agricultural Research Corporation, Embrapa Trigo, Passo Fundo, Brazil; ^3^ Agricultural and Biological Engineering Department, University of Florida, Gainesville, FL, United States; ^4^ Sustainable Agrifood Systems Program, International Maize and Wheat Improvement Center (CIMMYT), Texcoco, Mexico; ^5^ Institute for Sustainable Food Systems, University of Florida, Gainesville, FL, United States; ^6^ Regional Station, Bangladesh Wheat and Maize Research Institute, Rajshahi, Bangladesh; ^7^ Institute of Technology, Graduate Program in Applied Computing, University of Passo Fundo, Passo Fundo, Brazil; ^8^ Independent Consultant, Stuttgart, Germany; ^9^ Soil Science Division, Bangladesh Wheat and Maize Research Institute, Dinajpur, Bangladesh

**Keywords:** *Magnaporthe oryzae* pathotype *Triticum*, *Pyricularia oryzae*, biotic stress induced yield loss, process-based coupling, DSSAT, gridded crop modeling

## Abstract

Wheat is a crucial staple crop in South Asia and faces increasing risks due to interconnected agronomic and climate-related pressures. Wheat blast, caused by *Magnaporthe oryzae* pathotype *Triticum* (MoT), presents a persistent threat to wheat production in the region. This study evaluates its impact by analyzing the effects of sowing dates and wheat varieties on irrigated wheat grain yield in Bangladesh, where MoT was first identified in South Asia. A generic disease model (GDM), parameterized to reflect the disease’s characteristics, was used to simulate wheat blast inoculum build-up. The GDM incorporates temperature, relative humidity, and precipitation data to model the fungal life cycle and disease progression. The wheat crop simulation model, DSSAT-Nwheat, was integrated with the GDM to simulate MoT’s life cycle. This coupled model has been embedded into the Geospatial Crop Modeling and Decision Support Tool (GSSAT) to enhance agricultural decision-making. Using a primary dataset for validation and NASA Power reanalysis weather data, the simulated effects of wheat blast on wheat grain yield were analyzed across five sowing dates and four varieties in Bangladesh over a 23-year period from 2001 to 2023. The results indicate that late sowing leads to lower yields and higher disease incidence due to increased atmospheric moisture and temperature. Both model simulations and primary data demonstrated that varietal resistance to wheat blast can significantly mitigate yield losses of wheat. However, in southern Bangladesh, where weather conditions favor the disease, even the most resistant variety, BARI Gom 33, showed yield reductions resulting from wheat blast. These findings highlight the need for long-term breeding programs to develop cultivars suited to hot, humid conditions with high disease pressure, alongside short-term agronomic practices that minimize disease risk through sowing in optimum dates and less susceptible cultivars in Bangladesh.

## 1 Introduction

Crop pests and diseases account for direct yield reductions of 20–40% in global agricultural output (Oerke, 2006; Savary et al., 2012). Yet, their role in food security remains largely underappreciated, despite the significant impact of plant diseases on human livelihoods throughout history (Flood, 2010). Among these threats, wheat blast, caused by *Magnaporthe oryzae* pathotype *Triticum* (MoT), poses a particularly severe challenge to wheat production.

First identified in six municipalities in Paraná, Brazil, in 1985 (Igarashi et al., 1986), wheat blast affects all parts of the wheat plant, with MoT infection of wheat spikes leading to substantial yield losses (Cruz and Valent, 2017). Yield reductions are most severe when infection occurs during flowering and early grain formation stages (Goulart et al., 2007; Malaker et al., 2016). Following the initial appearance of the disease in Brazil, wheat blast spread to Bolivia, Paraguay, and parts of Argentina (Duveiller et al., 2016; Singh et al., 2021). In 2016, MoT unexpectedly appeared in South Asia, causing losses on over 15,000 ha wheat land in Bangladesh (Malaker et al., 2016; Mottaleb et al., 2018; Islam et al., 2020). At that time, wheat was the second most widely grown cereal crop in Bangladesh. Sudden, large-scale infection sparked fears potential regional spread (Dueri et al., 2016; Chowdhury et al., 2017). Following the initial outbreak, wheat blast continued to reappear in Bangladesh with reduced incidence and severity. Prevailing climatic conditions and a significant reduction in wheat cultivation likely contributed to this decline; however, the threat persists (Islam et al., 2019; Bishnoi et al., 2021, 2022). Regional projections indicate that South Asian farmers could lose up to 1.77 million tons annually with light infections of just 10% (Mottaleb et al., 2018). In Zambia, wheat blast was first reported during the 2017–2018 growing season and has since remained a concern, posing a significant risk of yield losses and economic damage (Tembo et al., 2020).

Reducing wheat blast’s impact on yield remains challenging due to two key factors: (1) the limited availability of genetic resistance (Cruppe et al., 2019, 2023; Juliana et al., 2022; Hossain, 2022) and (2) uncertainty about the profitability of fungicides, which show inconsistent efficacy and yield response (Cruz et al., 2019; Nunes Maciel, 2011; Ascari et al., 2021; Ristaino et al., 2021; Roy et al., 2021), especially under high disease pressure (Krupnik et al., 2024). Wheat blast can cause severe yield losses, with some studies reporting reductions of up to 100% (Ceresini et al., 2019; de Campos Dianese et al., 2021). Its impact depends on infection severity and timing, wheat variety susceptibility, and the effectiveness of management strategies.

A 2NS translocation segment from *Aegilops ventricosa* provides moderate resistance to wheat blast in some genotypes (Cruz et al., 2016). However, during the 2015 epidemic in Bolivia, even the best available resistance failed to control the wheat blast disease (Vales et al., 2018). The Bangladesh Agricultural Research Institute (BARI), in collaboration with the International Maize and Wheat Improvement Center (CIMMYT), developed and released BARI Gom 33 in 2017, the first wheat variety in Bangladesh carrying the 2NS translocation, offering moderate resistance to wheat blast (Hossain et al., 2019). This variety also provides a 5–8% yield increase over popular varieties and is zinc-enhanced (Mottaleb et al., 2019; Hossain et al., 2019). In 2020, the Bangladesh Wheat and Maize Research Institute (BWMRI) released BWMRI Gom 2 and BWMRI Gom 3, both are tolerant to wheat blast (https://bwmri.gov.bd/#; accessed on 27 January 2025).

Bangladesh is located in Wheat ‘Mega Environment 5’, which are geographic regions with similar climatic conditions where specific wheat genotypes can be cultivated with consistent yield outcomes, in this case with high tropical rainfall, high humidity, and average minimum temperatures for the coolest quarter between 11°C and 16°C (Sertse et al., 2024). Wheat in Bangladesh is sown after monsoon rice from the end of autumn to the beginning of winter on well-drained highlands, medium highlands, and medium lowlands, where water recedes before November (Krupnik et al., 2015a). One of the most important factors restricting wheat production is the sowing date (Barma et al., 2019). During the brief winter season in Bangladesh, wheat yield declined by 1.3% for each day that sowing was delayed beyond November 30 (Krupnik et al., 2015b). Late sowing of wheat causes low-temperature stress at the germination to seedling stages, but terminal heat stress at the reproductive stage is most concerning (Hossain and Teixeira da Silva, 2013; Barma et al., 2019).

Crop simulation models have been developed to assess alternative management options for specific environments, such as sowing dates (Hussain et al., 2018; Jahan et al., 2018). When built using primary experimental data, these models become valuable tools for extending experimental findings to different years, management practices, and environments (Matthews et al., 2002). One widely used platform is the Decision Support System for Agrotechnology Transfer (DSSAT), which offers a comprehensive framework for evaluating management strategies (Choudhury et al., 2021). Supporting over 40 crop models, DSSAT simulates crop growth, development, and yield by integrating soil-plant-atmosphere management dynamics (Jones et al., 2003; Hoogenboom et al., 2019).

Plant disease simulation models serve as valuable tools for decision-making in disease control by simulating epidemic development based on primary inoculum levels, weather conditions, and the availability of susceptible host tissue. These models provide insights that help optimize management practices to limit crop damage and prevent disease-inflicted economic losses. Bregaglio and Donatelli (2015), for instance, designed a predictive model to track *Magnaporthe oryzae* pathotype *Oryzae* (MoO, or rice blast) inoculum development throughout the cropping season. By incorporating temperature and relative humidity, the model simulates conidiophore development on an hourly basis. Later, Fernandes et al. (2017) modified this model for MoT by accounting for weather variables specific to wheat blast, validating them in comparison to observational data from outbreaks in Brazil.

Among the models within the DSSAT platform, DSSAT-Nwheat (Kassie et al., 2016) was recently updated with pest coupling points to link the wheat model with pest and disease models (Berton Ferreira et al., 2021). Pequeno et al. (2024) used DSSAT-Nwheat, integrated with the basic wheat blast model developed by Fernandes et al. (2017) to evaluate the disease’s potential impact on global wheat production. Simulations under current and future climate scenarios projected an annual reduction of 69 million tons, representing a 13% decline in global wheat production by mid-century. Their findings also identified Bangladesh as a country at significant continued and future risk of wheat blast. Compared to Afghanistan, China, India, Myanmar, Nepal and Pakistan, Montes et al. (2022) also found that Bangladesh is particularly vulnerable to wheat blast; they also highlighted the need for detailed studies to determine how agronomic management in the form of earlier sowing to escape periods of high disease stress and/or the adoption of more resistant varieties can mitigate these effects. In response, we utilized data from four season-years of multi-environment sowing date and wheat variety trials to calibrate and evaluate the performance of the DSSAT-Nwheat model. We simulated the potential impact of wheat blast on yield of wheat varieties with varying levels of wheat blast resistance and different sowing dates for the major wheat growing areas of Bangladesh over the last 23 years. Our results offer insights to refine agronomic management and genotype selection strategies in Bangladesh, demonstrating how optimized sowing dates and resistant varieties under changing climatic conditions can support evidence-based decision-making for smallholder farmers to mitigate yield losses and strengthen wheat production resilience.

## 2 Materials and methods

### 2.1 DSSAT-Nwheat model

**Figure 1:**
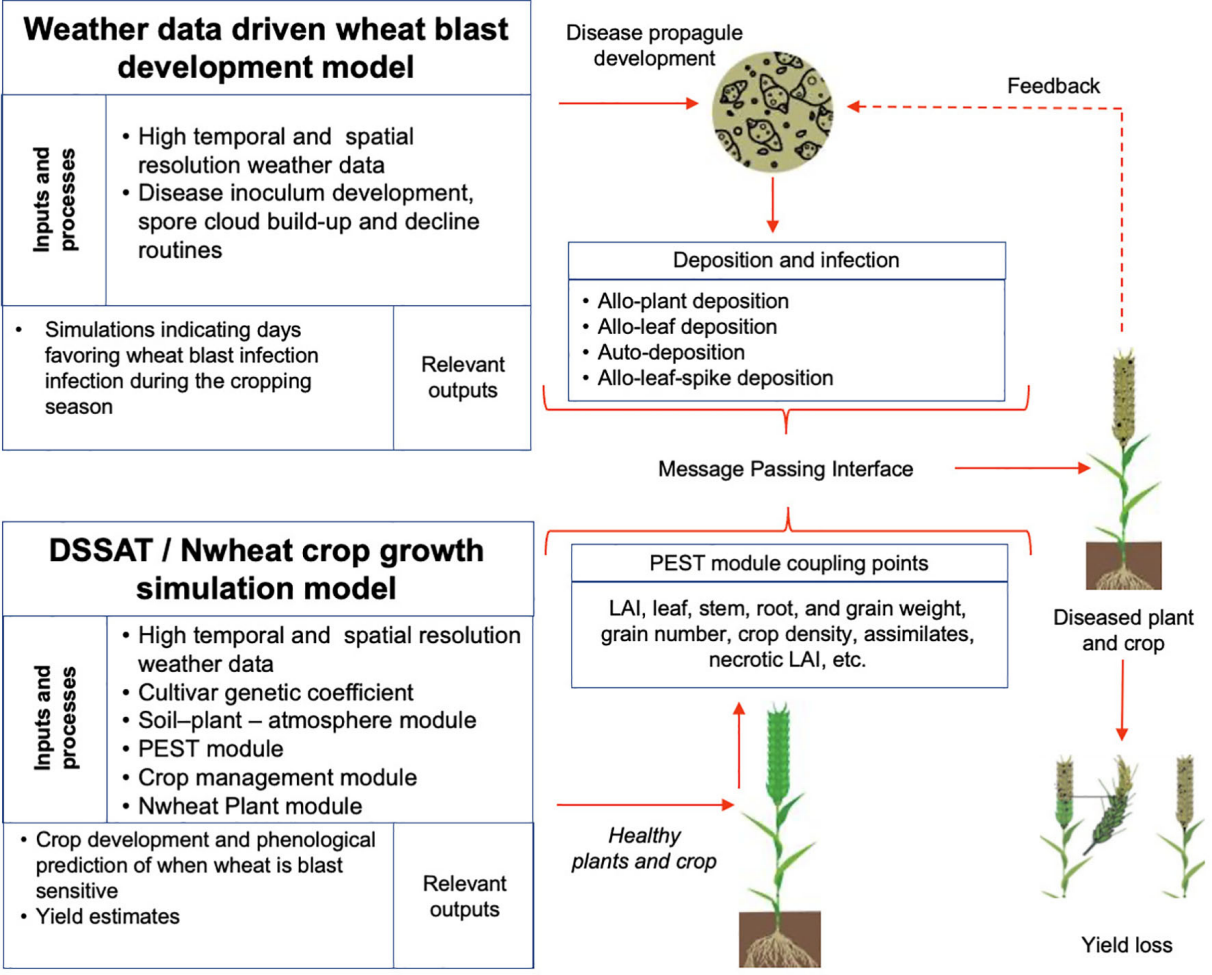
A simplified depiction of the integration of the DSSAT/Nwheat process-based crop model with a linked generic crop disease infection model that simulates yield losses resulting from wheat blast disease.

To generate actionable results, plant disease simulation models need to have a dynamic link with crop models (Bregaglio et al., 2016). DSSAT-Nwheat was recently enhanced with pest coupling points (**Figure 1**) which can be thought of as specific model variables whose changing values can be used to represent pest and disease damage to crop organs or growth processes. Plant disease simulators, on the other hand, are created to represent the impact on the final yield of a crop (Savary et al., 2006). The wheat simulation model used in this study was DSSAT-Nwheat, part DSSAT v.4.8.0.12 (Kassie et al., 2016). DSSAT is widely used in agricultural research, integrating data on soil, weather, crops, and management practices to predict crop growth, yield, and environmental impact. It has been applied across multiple regions and crops, supporting informed decision-making in agriculture under different climatic conditions. The model facilitates scenario analysis to assess the potential impacts of climate change, management strategies, and genetic improvements on crop productivity (Hoogenboom et al., 2019).

### 2.2 The generic disease model

The generic disease model (GDM) used in this study can be parameterized to simulate diseases across multiple crops (Fernandes et al., 2019). It comprises two components: a wheat-specific module developed in FORTRAN and a generic disease model coded in C++. The generic component parameterized for MoT simulates inoculum buildup and colonization of wheat spikes. As fungal mycelia spread, wheat grain development declines in proportion to disease intensity. The weather-based wheat blast simulator, first described by Fernandes et al. (2017), relies on hourly temperature, relative humidity, rainfall, and solar radiation data to generate predictions (**Figure 1**).

Weather-driven epidemiological models traditionally assess infection risk based on climatic conditions and estimate inoculum availability according to crop-specific characteristics. However, factors beyond seasonal climate, such as alternative hosts in the landscape, influence spore production by serving as inoculum reservoirs outside the wheat growing season (Pizolotto et al., 2019). The GDM incorporates these influences by simulating inoculum buildup at the beginning of the season, assuming the presence of alternative hosts before wheat reaches the grain-filling stage. A key assumption of the model is the uniform geographical presence of MoT inoculum across all simulated locations, without accounting for source-sink relationships or spore dispersal mechanisms.

The wheat blast prediction model, built on this framework, forms the core of the wheat blast disease early warning system (Krupnik et al., 2025), and consists of four components. The first component assumes the presence of sporulating wheat blast lesions in the environment and estimates conidiophore development rates based on temperature and relative humidity. These parameters are derived from earlier research by Bregaglio and Donatelli (2015) on rice blast disease. A generic disease simulator was first parameterized by Lazzaretti et al. (2016) to simulate the buildup of wheat blast inoculum and its subsequent colonization of foliage and wheat spikes. Building on this work, temperature and relative humidity are integrated to estimate blast inoculum potential for the hourly accumulation of inoculum potential and infection risk (Fernandes et al., 2017). To predict disease establishment and wheat blast damage rates during grain filling, the model simulates daily inoculum dynamics, including spore density within a 1 m³ volume above the canopy, survivability, and infection processes (Pequeno et al., 2024). The GDM code is licensed under BSD-3-Clause and is available upon request.

### 2.3 Crop and disease model coupling

The crop model coupling point concept was first introduced in 1983 (Boote et al., 1983). Several methodologies exist for coupling environmental models (Brandmeyer and Karimi, 2000), which can be adapted for crop and disease modeling. Variables such as leaf mass or area, stem mass, root mass, root length, and seed mass or number serve as coupling points that pests and diseases can adversely affect. By identifying mechanical and infection damage pathways, along with damage rates, growth models can be refined to assess their influence on crop development and yield. These considerations have been incorporated into the DSSAT crop modeling platform as a Pest Module (Batchelor et al., 1993; Jones et al., 2003).

The implementation of such models enables comprehensive analysis of key agricultural challenges, including in-season risk assessments and the impact of climate change and variability on crop growth and development (Bregaglio and Donatelli, 2015). For example, the DSSAT Pest Module allows users to input field observations or dynamically modeled data on insect damage, disease severity, and physical damage to plant components such as grains or leaves (**Figure 1**). These inputs facilitate simulations that estimate the potential impacts of pests and diseases on crop performance (Fernandes et al., 2019). To enhance model integration, different approaches have been explored, with message passing interface (MPI) methods emerging as a widely used solution (Pajankar and Pajankar, 2017). An MPI can be described as a standardized set of libraries for parallel and high-performance computing, consisting of exchanging messages between processes. In the case of the Pest Module, an MPI facilitates integration with pest and disease models, enabling dynamic simulations through the exchange of information between models (Browne and Wilson, 2015; Fernandes et al., 2019).

#### 2.3.1 Calibration and evaluation of DSSAT-Nwheat in Bangladesh

The calibration experiments were conducted at the Bangladesh Wheat and Maize Research Institute (BWMRI) in Dinajpur (23°11′14.52″ N; 89°11′11.99″ E; 10.4 masl), over four consecutive cropping seasons: 2017/2018, 2018/2019, 2019/2020, and 2020/2021. Evaluation experiments were carried out at the Rajshahi BWMRI station (24°22′ N; 88°39′ E; 20 masl) during the same cropping seasons. The same experiment was planted in each location, following a split-plot design with three replications.

The main plots included three sowing dates: November 25, December 15, and January 4 of each wheat growing season. The sub-plots contained wheat genotypes BARI Gom (meaning ‘wheat’ in Bangla) 26, BARI Gom 30, BARI Gom 32, and BARI Gom 33. Seeds were hand sown at a depth of 5–7 cm with 20cm row spacing following two tillage passes and covered with soil. The seeding rate was 120kg ha^-1^ for all varieties except BARI Gom 33, which tillers poorly and required an increased rate of 140kg ha^-1^, as recommended by BWMRI (2019). Fertilizers were applied at elemental rates of 100-27-40-20–1 kg ha^-1^ of N-P-K-S-B. Two-thirds of the nitrogen and all other fertilizers were applied basally, with the remaining nitrogen split applied before the first irrigation at crown root initiation (17–21 days after sowing (DAS)). Three light irrigations, each no more than 5cm deep, were applied: the first as described above, the second at booting (50–55 DAS), and the third at grain-filling (70–75 DAS). Weeds were managed to minimize competition. Dates for 50% anthesis and 80% maturity were recorded. After excluding 20cm borders to reduce edge effects, the remaining plot was harvested at physiological maturity and corrected to a grain moisture content of 12%. Canopy biomass was also measured from the same surface after sun drying to a constant weight. Further description of the experimental designs, crop management regimes, and data collection and analysis protocols, as well as comparisons between simulated and observed yields, are provided in **Supplementary Material 1**.

### 2.4 Gridded DSSAT-Nwheat

The DSSAT-Nwheat crop simulation model is part of the GSSAT system, a web-based platform for spatially explicit global crop simulations. GSSAT supports simulations across geographic areas and scenarios, such as crop management, fertilizer application, and initial conditions, by automatically retrieving weather data from NASA POWER and soil profiles from the Global High-Resolution Soil Profile Database for Crop Modeling Applications (Hoogenboom et al., 2019). Data and management practices are structured in a grid format to enable parallel execution and processing. Task pipelines facilitate parallel processing, enhancing simulation runtimes through simultaneous execution. Kubernetes handles container orchestration by automating deployment, scaling, and management of containerized applications across the cluster of machines., while DSSAT-Pythia, developed by the University of Florida, enhances efficiency by enabling parallel simulation execution (Hoogenboom et al., 2019).

### 2.5 Regional crop and disease simulations

**Figure 2:**
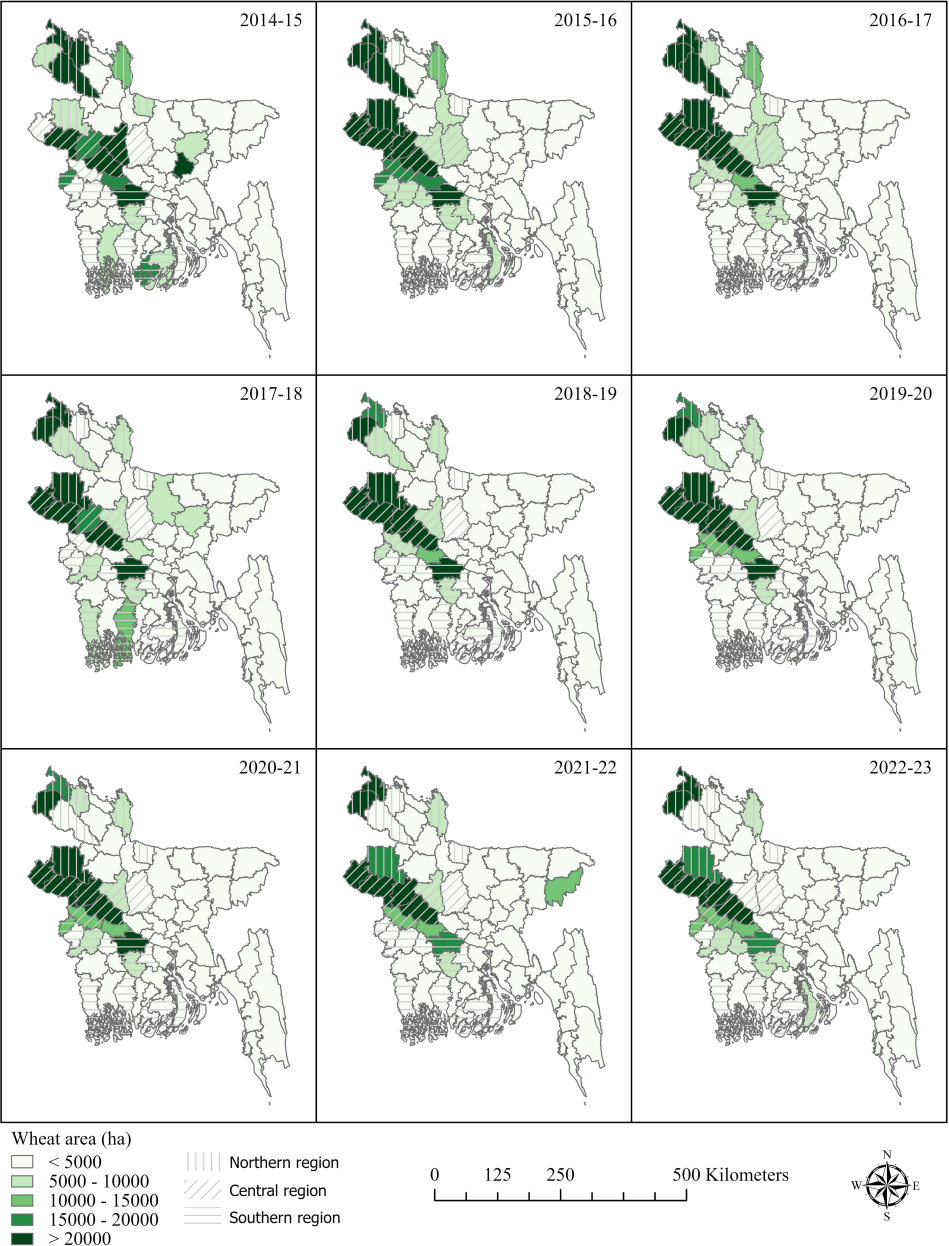
Maps of wheat cultivation areas in Bangladesh based on surveys by the Department of Agricultural Extension, illustrating spatial and temporal changes in wheat cultivation across districts from the 2014–2015 to 2022–2023 wheat growing seasons.

In general, plant disease models assume a uniform presence of inoculum (Bregaglio et al., 2016). The GDM, however, simulates spore survival, infection, and damage based on environmental conditions. All gridded crop modeling simulations were conducted on the HiPerGator high-performance computing cluster. Regional historical simulations were performed for 2001 to 2023: one with and one without disease damage, which allowed the calculation of the percentage loss attributed to wheat blast. We selected the growing seasons of 2015/2016 and 2020/2021 to compare yield losses from wheat blast, based on the historical observation of a severe epidemic in 2015/2016 and the notably low levels recorded in 2020/2021. To select a district for simulations, we focus on those with at least 5,000 hectares of wheat in any of the years surveyed (**Figure 2**), ensuring that our simulations target areas with significant wheat cultivation. We present our results based on three generalized regions of Bangladesh, defined by administrative districts where wheat cultivation met the minimum threshold during the 2021–2023 study period, using data from the Department of Agricultural Extension. These regions are the ‘northern’ (Thakurgaon, Panchagarh, Nilphamari, Kurigram, Dinajpur, Naogaon and Sherpur Districts), the ‘central’ (Nawabganj, Rajshahi, Natore, Pabna, Kushtia, Sirajganj, and Tangail Districts), and the ‘southern’ (Meherpur, Chuadanga, Jhenidah, Magura, Faridpur, Gopalganj, Madaripur, Bhola, Bagerhat, Patuakhali, Satkhira, and Barguna Districts).

### 2.6 Data processing and analysis

Districts in Bangladesh with a wheat-growing area of less than 5,000 hectares were excluded from this study to focus on regions with high production potential. The input files for each 0.1° x 0.1° grid cell in the three major wheat-growing regions included key parameters such as cultivar type, nitrogen application levels, sowing dates, irrigation practices, soil profiles, and local weather data. These comprehensive inputs were essential for capturing the variability across regions and simulating wheat growth accurately.

To assess yield vulnerability, the study calculated the absolute difference in grain yield between scenarios with and without disease effects. This metric offers insights into the potential economic and food security impacts of disease outbreaks across Bangladesh’s wheat-growing regions. The yield reduction due to the disease was expressed as a percentage loss following Equation 1 as follows,


(1) $$Yield\;Reduction\;\left(\%\right)=\left(1-\frac{Yd}{\left(Ynd\right)}\right)$$

where *Yd* is the grain yield under disease conditions, and *Ynd* is yield without the impact of the disease.

## 3 Results

This section of the paper is composed primarily of results from DSSAT-Nwheat simulations conducted over a 23-year period (2001–2023), capturing spatial and temporal variability in weather, wheat phenology, and yield performance under both wheat blast-infected and non-infected conditions. The results are organized in four parts: (1) temperature and rainfall patterns across regions and years, (2) phenological responses by sowing date and region, (3) simulated yield effects of sowing dates under disease and non-disease conditions, and (4) yield differences among varieties under epidemic and non-epidemic years. Unless otherwise indicated, results are derived from analysis of the 23-year simulation. When analyzing varietal susceptibility to wheat blast, we also present a focused comparison of two representative years—2016, when a major wheat blast outbreak occurred, and 2021, a season with notably low disease pressure—to highlight how varietal resistance performs under contrasting conditions.

### 3.1 Temperature and precipitation regimes

**Figure 3:**
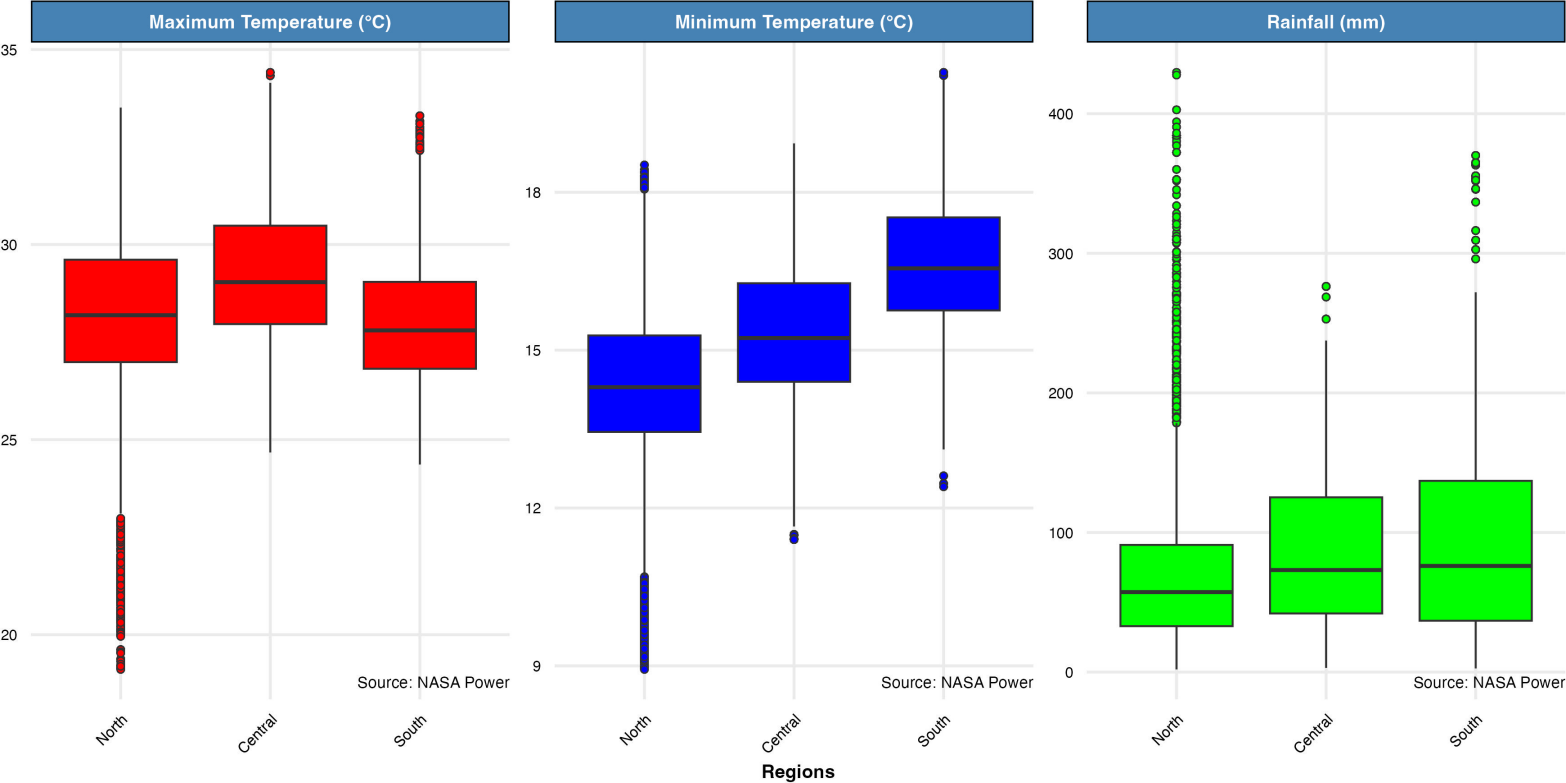
Box plots illustrating the maximum and minimum temperatures and rainfall variability during each simulation run from 2001 through 2023 grouped by regions of Bangladesh (North, Central, and South) generated with NASA Power data. Each box represents the interquartile range (IQR), with the central line indicating the median. Whiskers extend to values within 1.5 times the IQR, while points outside this range represent outliers.

Maximum temperatures from the 2021–2022 to 2022–2023 wheat cropping seasons in Bangladesh were relatively consistent across regions, ranging from 27.8 °C in the south to 29.1 °C in the central region (**Figure 3**). However, minimum temperatures showed greater variability, with the southern region experiencing the highest mean minimum temperature of 16.6 °C compared to 14.3 °C in the north. Lower rainfall was also evident in the northern compared to the southern region of the study area (**Figure 3**).

### 3.2 Regional differences in wheat phenology

**Figure 4:**
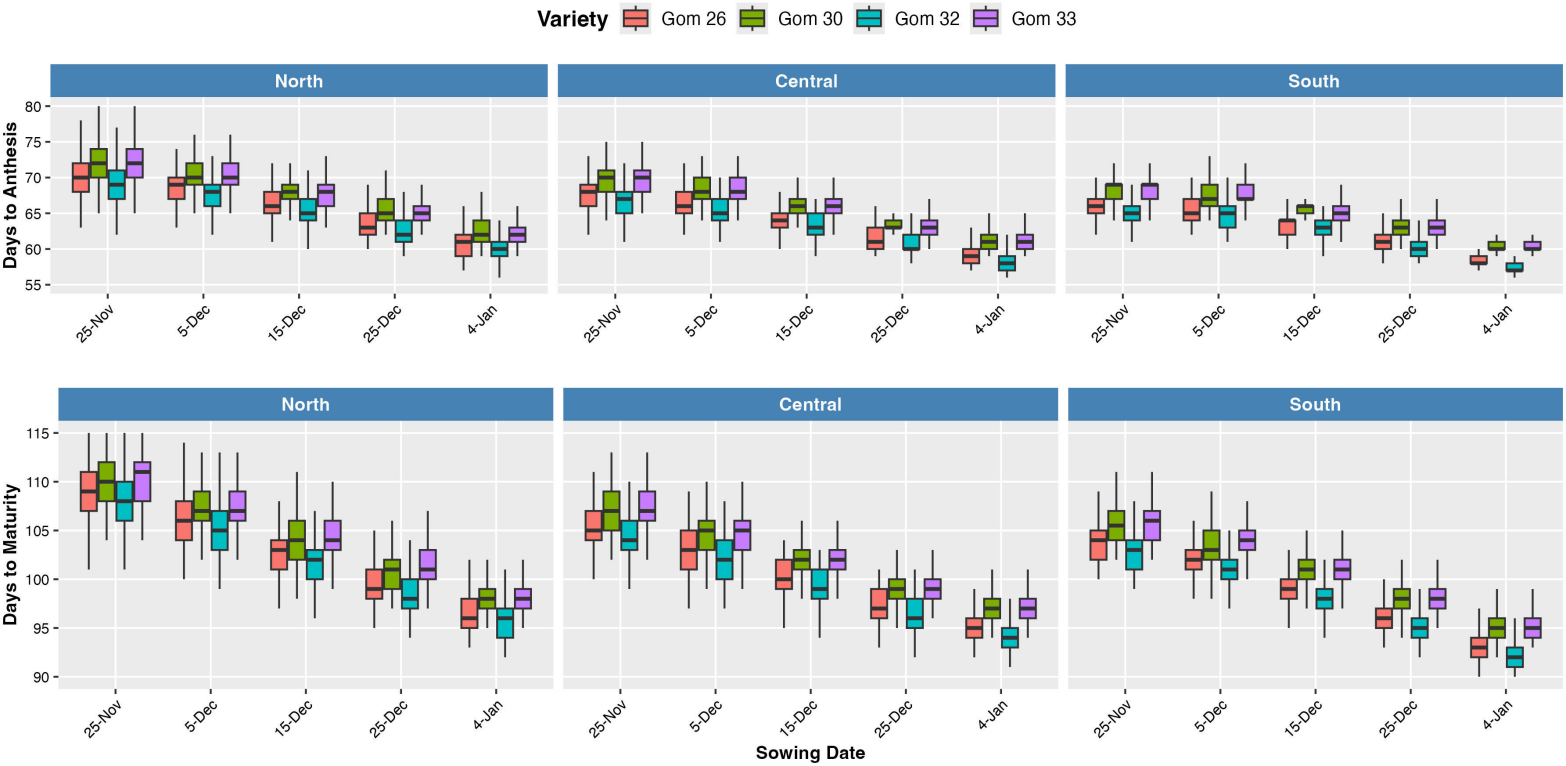
Box plots illustrating a simulated number of days from emergence to anthesis and from emergence to maturity across five sowing dates, aggregated across the full 2021–2023 period, grouped by regions of Bangladesh (North, Central, and South) and wheat variety. Each box represents the interquartile range (IQR), with the central line indicating the median. Whiskers extend to values within 1.5 times the IQR, while points outside this range represent outliers.

The phenological stages of wheat, including anthesis and physiological maturity, varied across the three wheat-growing regions and were influenced by sowing dates (**Figure 4**). The northern region exhibited a longer duration, with later anthesis and maturity, while the central and southern regions experienced progressively shorter phenological stages. Delayed sowing further influenced phenological development by reducing the time required to reach physiological maturity in the southern and central region.

### 3.3 Simulated impact of sowing dates on yield under wheat blast infected and non-infected conditions

**Figure 5:**
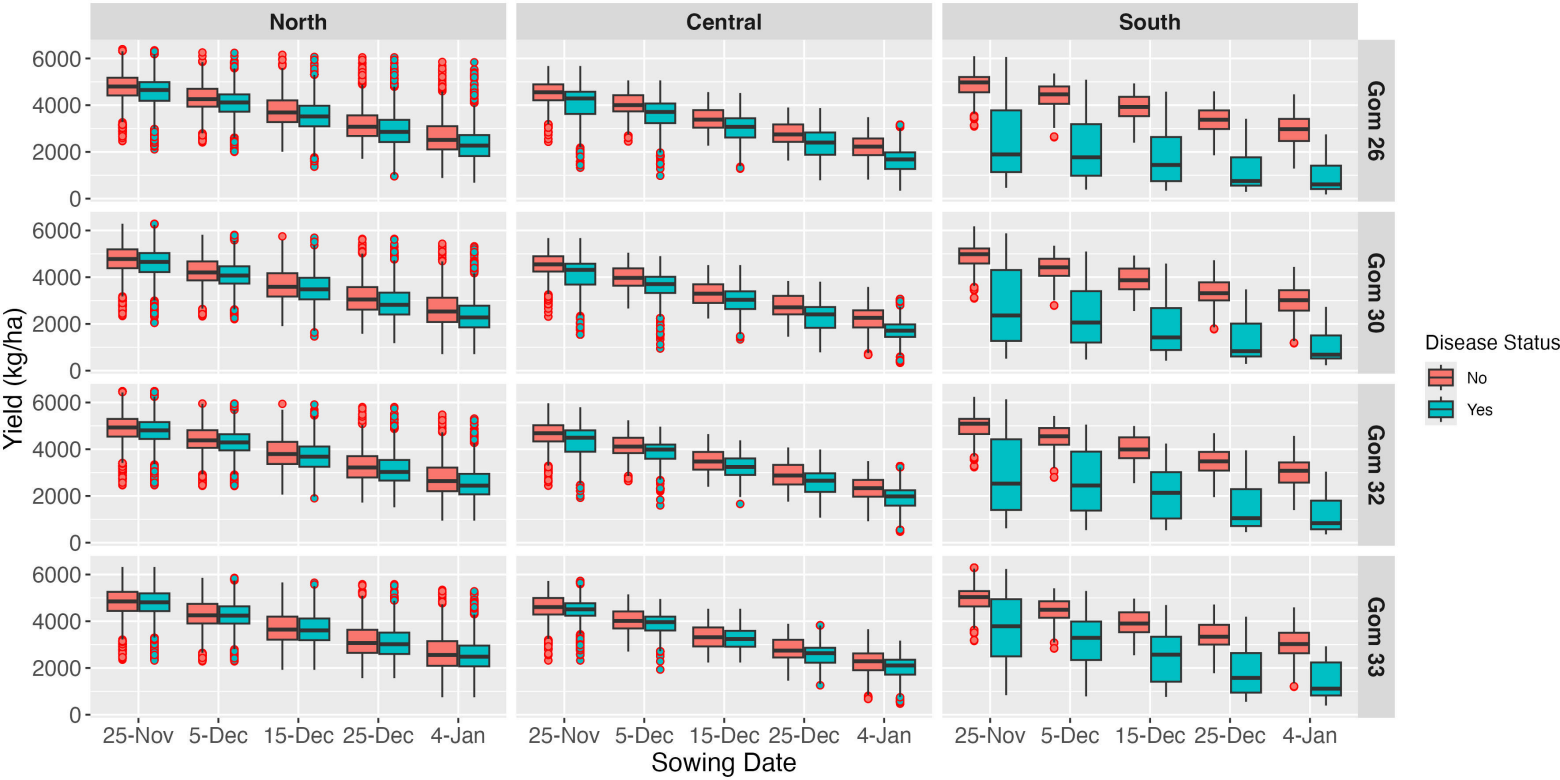
Box plots illustrating simulated wheat yield variability (kg/ha) across sowing dates, grouped by regions and variety, under two conditions: with and without disease. Each box represents the interquartile range (IQR), with the central line indicating the median. Whiskers extend to values within 1.5 times the IQR, while points outside this range represent outliers.

Simulations of wheat yield over a 23-year period (2001–2023) revealed a significant interaction between sowing dates and wheat blast impact (**Figure 5**). Five sowing dates were simulated at 10-day intervals from November 25 through January 4 (i.e., November 25, December 5, December 15, December 25, and January 4), allowing analysis of the effects of progressive delays in planting on phenology, yield, and wheat blast exposure. Delayed sowing, after November 25 in 10-day intervals, led to a marked decline in yields across all regions and scenarios. Notably, yield reductions associated with delayed sowing appear to be further exacerbated by wheat blast, which has increased rates of sporulation and infection risk as temperature rises during the course of the growing season.

The most severe yield penalties were observed in the southern region, which also had the lowest baseline yields. In contrast, the northern region consistently produced the highest yields, followed by the central region, highlighting regional differences in yield potential and vulnerability to wheat blast. The detrimental effects of delayed sowing were evident even in wheat blast-free scenarios. Furthermore, under simulated wheat blast conditions, these adverse effects were exacerbated, with additional yield loss.

### 3.4 Varietal susceptibility to wheat blast

**Figure 6:**
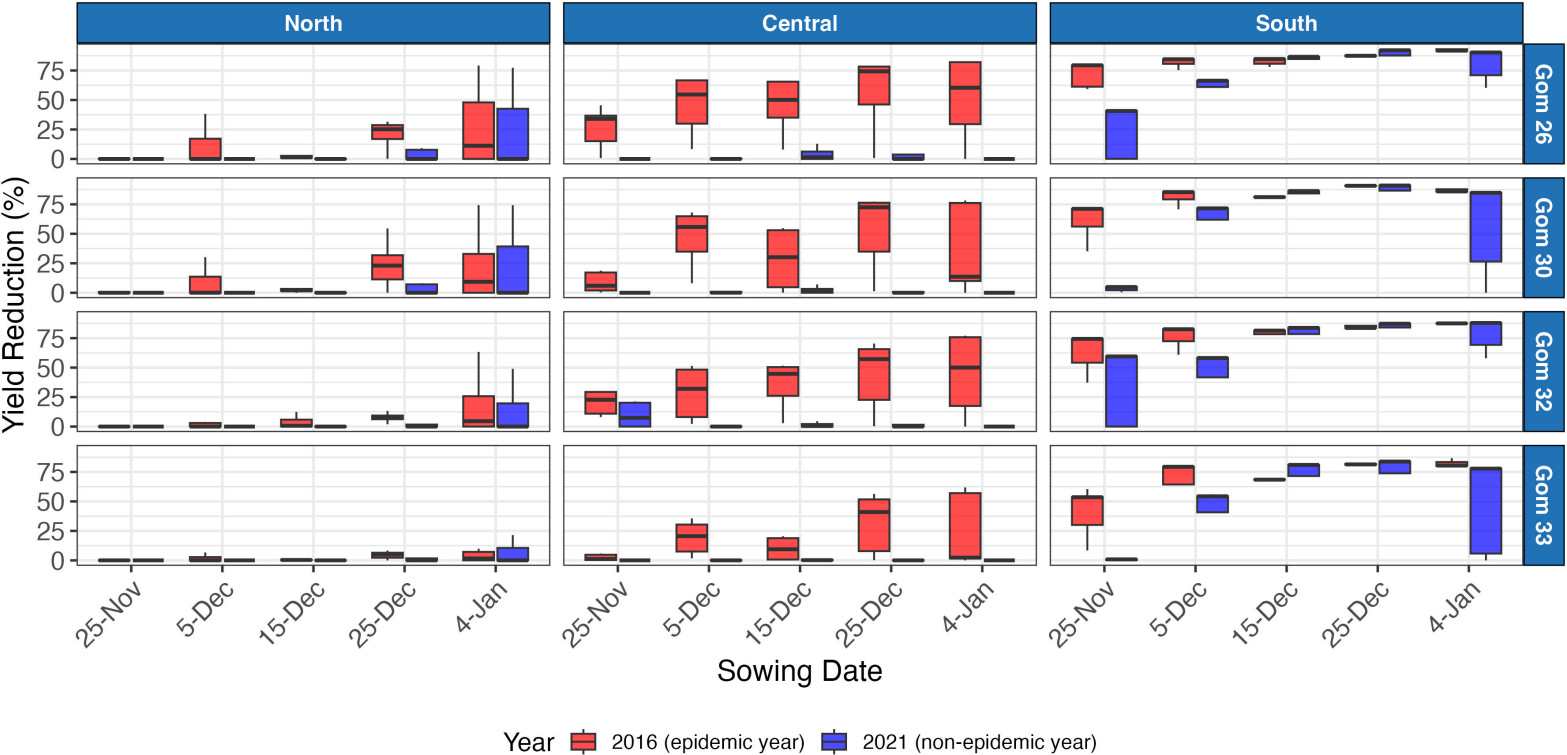
Simulated yield reductions for the four wheat varieties under a subset of years comparing epidemic (2016) and non-epidemic (2021) conditions across regions in Bangladesh. Each box represents the interquartile range (IQR), with the central line indicating the median. Whiskers extend to values within 1.5 times the IQR, while points outside this range represent outliers.

Across all regions and sowing dates, the northern region exhibited the least yield reduction. The central region experienced intermediate losses, while the southern region was the most severely affected (**Figure 6**). The simulated yield reductions varied significantly among wheat varieties, reflecting differences in their resistance to wheat blast. Highly susceptible varieties, such as BARI Gom 26, experienced the most substantial yield losses under high disease pressure, with simulated average yields of 920 kg ha^-1^ and minimum yields dropping as low as 179 kg ha^-1^. n contrast, the resistant variety BARI Gom 33 showed higher resilience, with simulated average yields of 1,459 kg ha^-1^ under high disease pressure, and minimum yields of 392 kg ha^-1^ in the most affected cases. These results emphasize the importance of deploying resistant varieties as a key management strategy for mitigating wheat blast impacts, particularly in the most vulnerable region.

## 4 Discussion

Simulations of wheat yield in the period between 2001 and 2023 showed large variation between sowing dates and regions in Bangladesh. The first sowing date simulated, 25 November, resulted in the highest wheat yield for all locations and varieties across study years. The sowing window for wheat in Bangladesh has tended to be recommended as between 15 and 30 November (Jahan et al., 2018), although in practice, and particularly in Southern Bangladesh, sowing after this period is common (Krupnik et al., 2015a). The timing of harvest of the previous rice crop and vacation of monsoon season floodwaters from fields is a major factor contributing to the timing of wheat sowing decisions, which often prevents early sowings (Krupnik et al., 2015b).

Across regions, varieties, and years studied, our simulation results suggested that delayed sowing resulted in severe yield decreases. The simulated yield decline per day of delayed sowing was 58.4 kg ha^-1^ in the northern region, 58.7 kg ha^-1^ in the central region, and 50.3 kg ha^-1^ in the southern region. Although the absolute daily yield reduction was lower in the southern region, this is largely due to its already lower baseline yields, which amplify the relative impact of disease pressure and environmental stress. The smaller rate of decline may also be partially explained by region-specific moderating factors, such as reduced maximum temperatures, increased rainfall, and longer day lengths, which could have buffered further losses under delayed sowing. Despite this, the southern region experienced the highest overall yield reductions and remained the most vulnerable to wheat blast in the simulations. In the absence of wheat blast, differences in wheat yield between sowing dates and regions can be partially attributed to variations in the maximum temperature recorded for simulation runs corresponding to each year, sowing date, variety, and grid cell. Yields were highly correlated (*R* = -0.87) to maximum temperature regimes on a daily basis for each sowing date and the corresponding growing period, variety, and grid cell simulated, which supports the findings reported by Thakur et al., 2018. Wheat is highly sensitive to heat, especially during the reproductive stage when terminal heat stress can interrupt pollination and grain formation (Hossain et al., 2019) and in Bangladesh, maximum temperature has been shown among the key weather parameters having a considerable effect on wheat yields (Amin et al., 2015). Furthermore, high temperatures can lead to shortened crop durations, which is also correlated with lower yields (Jahan et al., 2018), because of a decrease in the duration of the grain filling period (Sattar et al., 2023).

In line with these results, future climate change is expected to pose significant challenges to wheat production in Bangladesh (Rahman et al., 2018), adding to the country’s existing vulnerability to extreme weather events and climatic variability (Alam et al., 2023). As wheat production is highly sensitive to temperature (Asseng et al., 2011; Fischer et al., 2022), rising temperatures shorten the growing period, reduce grain yield, and ultimately threaten the sustainability of wheat production systems (Hossain and Teixeira da Silva, 2013). A study analyzing climate change effects between 1972 and 2010 found that increasing maximum temperatures had already significantly reduced wheat yields (Amin et al., 2015). However, our analysis from 2001 to 2023 did not identify significant temperature trends, likely due to the shorter observation period. Notably, no prior study has examined the combined effects of weather parameters, agronomic management, and wheat blast-related yield risks in Bangladesh.

Our results demonstrated substantial variation in yield and yield reductions across sowing dates and regions. While **Figure 6** presents a comparison of two representative years—2016 and 2021—to illustrate differences under epidemic and non-epidemic conditions, all other results in this study are based on the full 23-year simulation dataset (2001–2023). Higher minimum temperatures in the south and central regions created favorable conditions for wheat blast development, leading to greater yield losses. Conversely, lower minimum temperatures in the northern region appeared to restrict disease progression, resulting in smaller yield penalties. The lower wheat blast impact observed in the north may also be attributed to reduced rainfall during the wheat-growing season. Wheat blast thrives in warm, humid conditions, particularly when prolonged leaf wetness coincides with critical crop development stages (Krupnik et al., 2025; Pequeno et al., 2024). Our model suggests that lower rainfall reduced leaf wetness duration, limiting disease infection and spread, while higher rainfall in the central and southern regions created more favorable conditions for disease development. However, it is important to note that delayed sowing itself also likely contributed to lower yields, as indicated in prior studies in Bangladesh (Krupnik et al., 2015a, b), independent of disease pressure. The greatest losses occurred when late sowing coincided with environmental conditions highly conducive to wheat blast, indicating a compounded effect of agronomic and disease-related stressors.

The impact of wheat blast was further exacerbated by late-season planting across Bangladesh’s wheat-growing areas, resulting in lower yields. Yield losses were most pronounced in the south, where simulations indicated a mean reduction of 51%, compared to 11% in the central region and 5% in the north. These differences can be partly attributed to higher temperature and humidity levels in the south, which drive inoculum buildup (Fernandes et al., 2017). Additionally, higher temperatures during later growth stages accelerated crop development and the number of days to maturity (data not shown), reducing growth duration and increasing susceptibility to wheat blast, particularly during anthesis and early grain filling. The greatest losses occurred when late sowing coincided with environmental conditions highly conducive to wheat blast, indicating a compounded effect of agronomic and disease-related stressors.

Although absolute daily yield declines were slightly lower in the southern region (47 kg ha^-1^ day^-1^) compared to the northern (56 kg ha^-1^ day^-1^) and central (61 kg ha^-1^ day^-1^) regions, this is explained by already lower baseline yields in the south due to higher disease pressure and suboptimal growing conditions, the latter described in detail by Krupnik et al. (2015a, b). When accounting for total yield losses, the southern region remained the most affected among the regions studied, with late sowing amplifying the risk of yield reductions. MoT infection of spikes remains the primary cause of yield losses (Fernandes et al., 2017), leading to partial or total sterility (Cruz and Valent, 2017). Delayed sowing increases stress due to higher temperatures during late phenological stages, which directly affects grain filling and indirectly heightens blast pressure.

A major constraint for wheat production in Bangladesh is the narrow sowing window (Krupnik et al., 2015a, b). A recent study found that 84% of wheat farmers in northwest Bangladesh observed a shortening of the winter season over the past two decades (Tasnim et al., 2023). Farmers responded by shifting to alternative winter crops, such as maize and potato, rather than adjusting sowing dates or adopting more adaptable wheat varieties. However, the scope for sowing date adjustments remains limited by the timing of the preceding rice harvest, which is influenced by monsoon onset and farmers’ ability to drain fields for wheat sowing (Krupnik et al., 2015a).

Our simulations revealed a range of yield and disease-related responses among wheat varieties, underscoring the importance of genetic resistance in mitigating wheat blast impacts (Cruz et al., 2016). The highly susceptible variety BARI Gom 26 experienced severe yield losses under high disease pressure, whereas BARI Gom 33, which carries the 2NS translocation associated with partial resistance (Juliana et al., 2022; Krupnik et al., 2024), performed better under similar conditions. These findings align with previous studies highlighting the importance of deploying resistant cultivars in regions prone to wheat blast (Vales et al., 2018). However, even BARI Gom 33 experienced yield losses, suggesting that current resistance sources, including the 2NS translocation, may not provide complete protection against MoT under highly favorable weather conditions or pathogen evolution.

To address these challenges, efforts should focus on identifying durable wheat blast resistance genes beyond the 2NS translocation to counter evolving MoT isolates. Tosa (2021) identified key resistance genes that could be utilized in future breeding programs. Further studies are needed to understand how the genetic background of wheat varieties influences 2NS-based resistance under current and future weather conditions. Additionally, incorporating complementary genetic components to enhance 2NS effectiveness across different environments could improve resistance durability (Cruppe et al., 2019). Cross-border collaboration in exchanging resistant germplasm and disease monitoring data has proven valuable in these efforts (Singh et al., 2021).

Our calibration and evaluation of the DSSAT-Nwheat simulation model aligned outputs with observed data, enabling retrospective analysis and future scenario planning. Wheat phenology significantly influences crop development and grain yield (Ceglar et al., 2019). The model accurately predicted phenological stages such as anthesis and maturity, closely matching observed values (**Supplementary Materials 1**). The simulations quantified wheat yields over 23 years and provided insights into biotic and abiotic stress impacts, including disease outbreaks and their link to climate variability. This modeling approach offers valuable insights for policymakers to identify wheat blast-vulnerable areas and develop targeted interventions to enhance productivity in Bangladesh’s wheat-growing regions.

Considering short-term approaches to improving wheat production, our findings support earlier sowing as an effective strategy to enhance yields (Jahan et al., 2018; Krupnik et al., 2015a, b). Investments in EWS for predicting wheat blast outbreaks and guiding farmers in disease management have been proposed (Kim and Choi, 2020; Fernandes et al., 2021), and recent work has highlighted how EWSs for wheat blast have been scaled in Bangladesh and Brazil (Krupnik et al., 2025). However, like other diseases, effective implementation of EWS will require robust pathogen surveillance networks and real-time data-sharing platforms (Smith et al., 2024).

To advance our understanding, we translated the conceptual disease model into functional simulations within the DSSAT/Nwheat Pest Module. This integration allowed the evaluation of biotic stresses within the DSSAT-Nwheat model to assess wheat blast fungus dynamics under climate variability, which was previously limited to wheat rust (Caubel et al., 2017). However, our study has practical limitations. Weather-driven epidemiological models typically assess infection risk based on climate conditions while assuming inoculum is not a limiting factor (Magarey et al., 2005; Donatelli et al., 2017; Launay et al., 2020). The assumption that wheat blast can persist on alternative hosts, such as grasses, outside the main growing season needs further validation through regional studies in South Asia, this assumption influences wheat disease development (Danelli et al., 2019; Vicentini et al., 2023), and hence model performance.

Another limitation relates to irrigation practices, which create humid microenvironments conducive to MoT growth and spread. Currently, the DSSAT/Nwheat Pest Module does not incorporate detailed irrigation sub-routines considering application timing, method (e.g., flood, furrow, or drip), and soil moisture retention effects on crop microclimate. Although our simulations were conducted for irrigated wheat, the lack of such sub-routines highlights the need for further refinements to better capture irrigation-driven humidity dynamics. Additionally, uncertainties associated with soil profile data from the Global High-Resolution Soil Profile Database for Crop Modeling Applications, used as inputs for the DSSAT-Nwheat model, may impact simulation accuracy. While these datasets provide valuable large-scale soil information, they may lack the resolution needed to capture field-level variability. These uncertainties, coupled with potential errors in NASA POWER weather data, could influence the reliability of simulated wheat blast risk and yield outcomes. Future research should incorporate local soil measurements and observed weather data to enhance model calibration and validation.

## 5 Conclusions

In this study, we coupled the DSSAT-Nwheat crop growth simulation model with a generic disease model (GDM) to assess the effects of sowing dates and wheat varieties on irrigated wheat grain yield with and without wheat blast disease over a 23-year period in Bangladesh. Our results indicate that late sowing increases yield losses and disease incidence due to unfavorable growing conditions and a more conducive environment for disease development, particularly in southern Bangladesh. While resistant varieties like BARI Gom 33 helped reduce losses, they remained vulnerable under high disease pressure and suboptimal planting times. These findings highlight the dual importance of early sowing and genetic resistance in managing wheat blast risks.

Our simulations validate the DSSAT-Nwheat model’s ability to reproduce wheat growth and yield patterns under variable environmental and disease conditions, offering the first long-term, region-specific analysis of wheat blast impacts in South Asia. Our modeling results underscore how phenology, temperature, and relative humidity interact to shape disease pressure and yield outcomes under wheat blast disease. These insights can support farmer decision-making, especially in areas where timely sowing is constrained by rice harvest timing that may delay establishment of the subsequent wheat crop. The persistence of yield losses even in resistant varieties points to the urgent need for breeding programs that ensure durable sources of resistance. The study also reinforces the value of coupled crop–disease models in supporting early warning systems that combine real-time weather data with disease surveillance. While grounded in the Bangladesh context, the modeling framework has direct applicability in other blast-prone regions such as Zambia and South America. It provides a transferrable approach to evaluating climate–disease–management interactions and improving resilience in wheat production systems.

## Data Availability

The datasets presented in this study can be found in online repositories. The names of the repository/repositories and accession number(s) can be found in the article/**Supplementary Material**.

## Supplementary material

The **Supplementary Material** for the manuscript is available online in R-Markdown format at https://github.com/thiagoferreira53/paper-Nwheat-GDM-Bangladesh. The document describes a multi-environment and varietal trial carried out during the wheat-growing seasons of 2017/2018, 2018/2019, 2019/2020 and 2020/2021 in the locations of Dinajpur and Rajshahi in Bangladesh. The experimental design is also presented. The dataset was used to calibrate and evaluate genetic-specific parameters for the wheat varieties BARI Gom 26, BARI Gom 30, BARI Gom 32, and BARI Gom 33. The dataset includes information on soil characteristics, daily weather data, and crop phenology and growth observations measured during the experimental trials. Model performance was assessed using multiple goodness-of-fit metrics by comparing the simulated results against the field observations for each genotype sowing date combination.
